# La ruta abreviada de comparabilidad colombiana para medicamentos biotecnológicos: ¿tendencia global o caso único?

**DOI:** 10.26633/RPSP.2017.94

**Published:** 2017-09-11

**Affiliations:** 1 Facultad de Medicina Universidad de Antioquia Antioquia Colombia Facultad de Medicina, Universidad de Antioquia, Medellín, Antioquia, Colombia; 2 Facultad de Medicina Universidad de Antioquia Antioquia Colombia Facultad de Medicina, Universidad de Antioquia, Medellín, Antioquia, Colombia

*Estimado editor*,

Hemos leído el informe “El debate de la regulación de medicamentos biotecnológicos: Colombia en el contexto internacional” (1). El artículo inicia describiendo la regulación de precios de medicamentos basada en referencias nacionales e internacionales como una estrategia de “alivio fiscal parcial al sistema de salud colombiano”, y resalta que solo mediante la competencia esta estrategia sería completa y duradera. La figura 1 ilustra, sin lugar a dudas, el impacto positivo en la reducción de precios (-23%). En el resto del artículo se busca justificar la llamada “ruta abreviada de la comparabilidad colombiana” (RACC), que elimina la obligatoriedad de estudios preclínicos y clínicos, y se concluye que se “evidencia que dicha posición no es aislada y está basada en tendencias regulatorias globales”. Lamentamos estar en desacuerdo. La información contenida en el mismo estudio y otras referencias nos llevan a una conclusión diferente.

No es lo mismo eximir que reducir. Aunque los autores usan el término exención en forma permanente, por ejemplo, “en la ruta abreviada colombiana no se permite exenciones para los estudios analíticos, las posibles exenciones se refieren exclusivamente a los estudios clínicos o preclínicos”; en la conclusión modifican el término y sostienen que la RACC solo implica “reducción de pruebas clínicas”, lo cual seguiría la tendencia mundial.

Según el cuadro 3 del artículo, Brasil acepta la reducción (no exención) de estudios preclínicos y fase I–II pero exige fase III; Ecuador exige estudios clínicos fase I–II; México exige estudios preclínicos y clínicos; en Chile también se contempla una posible reducción (no exención); en Argentina el ejercicio de comparabilidad se acompaña de estudios preclínicos y clínicos, cuya profundidad y amplitud dependen de la caracterización del producto; y en Uruguay los requerimientos se evalúan caso por caso (la exención es posible). Entonces, la ruta de comparabilidad, sin exenciones, es la normal en cinco de los seis países (83%); no obstante, los autores concluyen que “el decreto colombiano incorpora los mismos requisitos considerados en otras regulaciones para exceptuar los experimentos clínicos confirmatorios”. Dicha conclusión no se deriva de los datos presentados.

La mencionada facultad discrecional de la Administración de Medicamentos y Alimentos de los Estados Unidos (Food and Drug Administration, FDA) es diferente a la posición reglada de la exención sistemática, “explícita y visible” de estudios en la RACC. India, país en desarrollo con más de 1 000 millones de habitantes, exige estudios comparativos preclínicos y clínicos (farmacocinéticos y farmacodinámicos) (2); y afirma que la exención de estudios clínicos de eficacia y seguridad (fase III) dependerá de cumplir cuatro condiciones: la comparabilidad estructural y funcional se puede determinar *in vitro* con un alto grado de confianza; el biológico similar es comparable al producto de referencia en todas las evaluaciones preclínicas (se exime en la RACC); la comparabilidad farmacocinética (FC) y farmacodinámica (FD) ha sido demostrada en pacientes, incluida la inmunogenicidad (FC/FD se exime en la RACC); y el fabricante presenta un plan de manejo de riesgo con énfasis en la inmunogenicidad. Al contrario de las conclusiones de los autores, esto refuerza que la exención completa no es una “tendencia regulatoria global”.

Independiente del riesgo sobre la salud, los autores describen como argumentos adicionales para la exención de estudios de comparabilidad: el mayor costo y tiempo para hacer esos estudios respecto a las pruebas para genéricos y la dificultad técnica y metodológica, especialmente en enfermedades raras. Como bien se evidencia en el manuscrito en el cuadro 1, las ventas de biológicos son altísimas, por lo que la inversión de la industria privada en biocompetidores estará justificada en una buena probabilidad de retorno, sin mencionar que la exención va en contra del ya mermado y desfinanciado sistema nacional de ciencia y tecnología colombiano (3). Para los investigadores, la larga duración y la dificultad metodológica son retos diarios.

Los autores hacen una afirmación contundente: “los avances científicos y el conocimiento cada vez mayor sobre las proteínas permiten superar el paradigma de la comparabilidad”; sin embargo, no está explicada ni soportada por ninguna referencia, por lo cual es solo una opinión personal y no constituye un argumento científico.

Finalmente, la posición de la Organización Mundial de la Salud (OMS) no se modificó con la Resolución WHA 67.21, de hecho se fortaleció según el anexo 3 del sexagésimo sexto reporte del Comité de Expertos en Estandarización Biológica (4). En la página 135 de este reporte se afirma que, si a lo largo de todo el proceso de desarrollo no se realiza una comparación del producto biológico biosimilar con el de referencia, no puede denominarse biosimilar, e insiste en que no es un medicamento genérico. Además, se señala que hay países donde se registraron medicamentos biológicos sin seguir el estándar de comparabilidad, como Colombia, e incluso se han registrado como genéricos. En consecuencia, la OMS insta a las agencias reguladoras nacionales a que identifiquen estos productos e inicien con los fabricantes un proceso gradual de ajuste para que cumplan los requerimientos actuales. Estas disposiciones no fueron mencionadas o aceptadas por los autores.
